# Oligomerization of Human Cystatin C—An Amyloidogenic Protein: An Analysis of Small Oligomeric Subspecies

**DOI:** 10.3390/ijms232113441

**Published:** 2022-11-03

**Authors:** Daria Wojciechowska, Michał Taube, Karolina Rucińska, Joanna Maksim, Maciej Kozak

**Affiliations:** 1 Department of Biomedical Physics, Faculty of Physics, Adam Mickiewicz University, 61-614 Poznań, Poland; 2 National Synchrotron Radiation Centre SOLARIS, Jagiellonian University, 30-392 Kraków, Poland

**Keywords:** human cystatin C, oligomer, amyloid, small-angle X-ray scattering, dynamic light scattering

## Abstract

Human cystatin C (HCC), an amyloidogenic protein, forms dimers and higher oligomers (trimers, tetramers and donut like large oligomers) via a domain-swapping mechanism. The aim of this study was the characterization of the HCC oligomeric states observed within the pH range from 2.2 to 10.0 and also in conditions promoting oligomerization. The HCC oligomeric forms obtained in different conditions were characterized using size exclusion chromatography, dynamic light scattering and small-angle X-ray scattering. The marked ability of HCC to form tetramers at low pH (2.3 or 3.0) and dimers at pH 4.0–5.0 was observed. HCC remains monomeric at pH levels above 6.0. Based on the SAXS data, the structure of the HCC tetramer was proposed. Changes in the environment (from acid to neutral) induced a breakdown of the HCC tetramers to dimers. The tetrameric forms of human cystatin C are formed by the association of the dimers without a domain-swapping mechanism. These observations were confirmed by their dissociation to dimers at pH 7.4.

## 1. Introduction

Human cystatin C (HCC), also known as cystatin 3, post-gamma globulin or formerly as human gamma trace (MW 13,335 Da, 120 amino acids in posttranslational form, UNIPROT entry: P01034), was characterized and identified by Grubb and co-workers as an important protein inhibitor of cysteine proteases [1,2,3].

**Figure 1:**
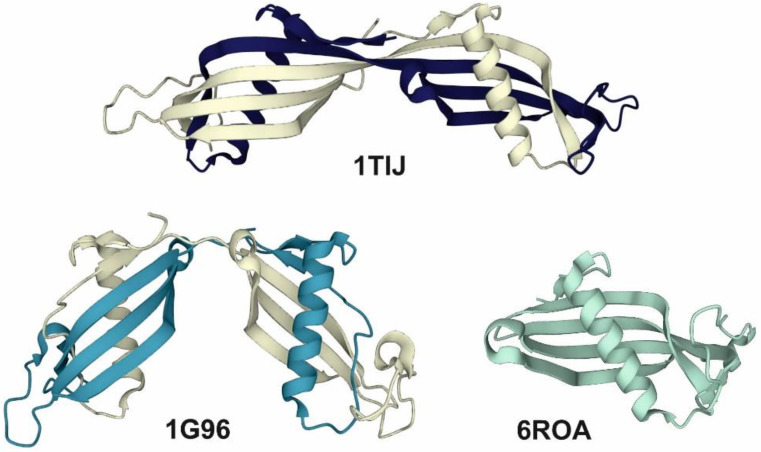
The structure of native HCC dimes in the open (PDB code: 1TIJ [9]) or partially closed (PDB code: 1G96 [5]) conformation and the V57G HCC monomer (PDB code: 6ROA [9]).

In its physiologically active form, this protein acts as a monomer [4], although its oligomerization ability via domain-swapping phenomena is well known [5,6]. As a result of this process, this protein forms dimers, trimers and different higher oligomeric species. However, the atomic structures of these species have been solved using X-ray crystallography only for the dimers of native or truncated HCC and several HCC mutants [5,7]. The crystal structure of the monomer was solved for mutants stabilized by an additional disulfide bridge (L47C-G69C) [8] or V57G mutation [9]. The dimerization ability mentioned above manifests itself not only in dimer formation through the domain-swapping mechanism, but also in the formation of a flexible dimer form in open [10] or partially closed [5] conformations (see Figure 1).

HCC is widely used in medical diagnostics as a useful marker of kidney dysfunction, in particular as a molecular detector of the so-called “shrunken pore syndrome” [11,12]. Moreover, this protein in its mutated form (L68Q) is associated with the occurrence of hereditary cystatin C amyloid angiopathy (HCCAA), also known as Icelandic-type amyloidosis or type VI amyloidosis (for review see: [13,14]), where HCC molecules build up fibrillary deposits in the blood vessels causing hemorrhages, typically gradually leading to the death of relatively young patients (20–30 years old), although the case of an elderly patient has also been recorded [14,15,16]. Human cystatin C is also associated with the occurrence of neurodegenerative diseases such as Alzheimer’s disease, as decreased HCC levels have been observed in patients with this disease, and it has also been found in the form of co-deposits in amyloid β plaques [17]. The aforementioned oligomeric forms of cystatin C (cyclic trimers, decamers and dodecamers) were previously characterized in our earlier papers [18,19,20]; however, all of these structures were created through the domain-swapping process.

The aim of our research was, therefore, to define the spectrum of conditions in solution under which HCC is capable of forming dimers, tetramers and possible higher oligomers. We also wanted to use complementary methods (DLS, SAXS, SEC) to determine the structural parameters or even reconstruct the structure of the HCC oligomers. The behavior of HCC under various pH conditions, with buffers and in the presence of factors supporting protein unfolding was of particular interest to us. Given the widespread use of HCC as a marker of kidney function, the determination of the conditions for the formation of oligomeric forms seems very useful and of great value.

## 2. Results

Our studies were carried out in two ways; the first series of experiments was focused on the behavior of human cystatin C under various pH conditions, and then the oligomerization tests of HCC dimers were performed. Finally, the stability and structure of the tetrameric forms of HCC were characterized.

### 2.1. Behaviour of Human Cystatin C under Various pH Conditions

**Figure 2:**
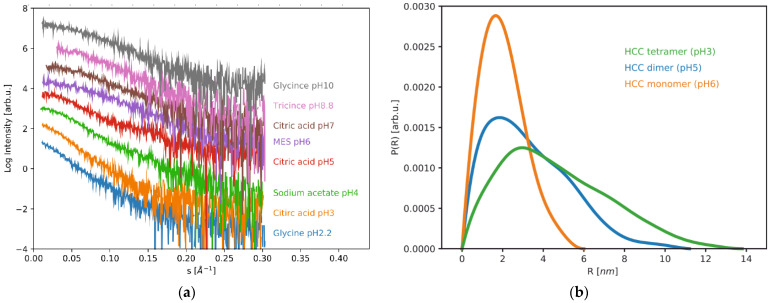
Solution scattering data collected for human cystatin C at different pH levels: (**a**) SAXS curves recorded for HCC in pH from 2.2 to 10; (**b**) pair distance distribution functions for HCC monomers (pH 6), dimers (pH 5) and tetramers (pH 3).

Biophysical studies of amyloidogenic proteins are very prone to error due to the inherent instability of such proteins. Even a minute amount of aggregates or seeds for aggregation can drastically change the output of the experiments. Therefore, it is very important to find the conditions for stable protein purification and storage and subsequent experiments. Sometimes the conditions that induce aggregation and fibrilization need to be optimized for the requirements of an experiment. For this reason, in the first part of our study, we analyzed the behavior of human cystatin C under different pH conditions (pH 2.2–10) in solutions imitating various body fluids and potential oligomerization buffers. In addition, we also analyzed the effects of gentle denaturing conditions (the presence of 0.2 M guanidinium chloride, GdmCl) on the ability to induce the transformation of HCC to higher order forms. To study the structure and oligomerization state of the HCC protein, we used the small-angle X-ray scattering (SAXS) technique, which is very useful for the characterization of the size and shape of the macromolecules, without strict requirements for the buffering solutions [21,22]. The SAXS data recorded for the HCC protein solutions in all of the mentioned pH conditions are presented in Figure 2a.

The radius of gyration (*R*_G_) is a useful parameter that describes the size of the molecule and can be used to track the changes of the oligomerization state or changes in shape and can be extracted from the SAXS data. The *R_G_* values of HCC in the studied conditions are presented in Table 1. A comparison of the distance distribution functions calculated for the HCC tetramer, dimer and monomer and the pH dependence of the radii of gyration are presented in Figure 2b.

From the shapes of the obtained SAXS curves and observed changes in the radii of gyration values, it is evident that human cystatin C changes its size or shape at acidic pH levels. In the acidic environment, the HCC samples were characterized by *R_G_* values of 3.9 nm and 3.42 nm at pH 2.2 and pH 3, respectively. The size of the HCC molecule (*D*_max_), calculated from the pair distance distribution functions, was 17 nm at pH 2.2 and at pH 3 it was 14 nm. These values indicate the presence of higher oligomers that are larger in size than the dimers. Upon increasing the pH to 4 and 5, we observed a decrease in *R_G_* and *D*_max_ values. At pH 4, *R_G_* was 2.64 nm and *D*_max_ was 12 nm, while at pH 5, *R_G_* was 2.4 nm and *D*_max_ was 11.2 nm. These values are in close similarity to the values obtained previously for a domain-swapped dimer of the HCC [23]. At pH 6, we observed the lowest values of both *R_G_* = 1.55 nm and *D*_max_ = 6 nm, indicating that at pH 6 human cystatin C exists in the monomeric form. With a further increase in pH values from 7 to 10, we observed slight increases in the *R_G_* and *D*_max_ values up to 1.73 nm and 7.8 nm, respectively, at pH 10.

**Figure 3:**
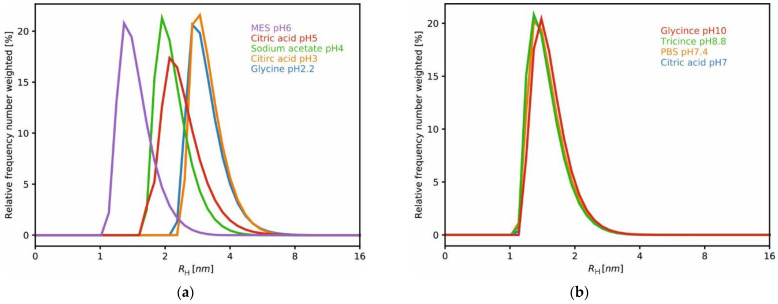
DLS data collected for human cystatin C at different pH levels: (**a**) DLS results—particle size distribution of human cystatin C (HCC) in various buffers; (**b**) pH dependence (range 2.2–6); (**b**) pH dependence (range 7–10).

**Figure 4:**
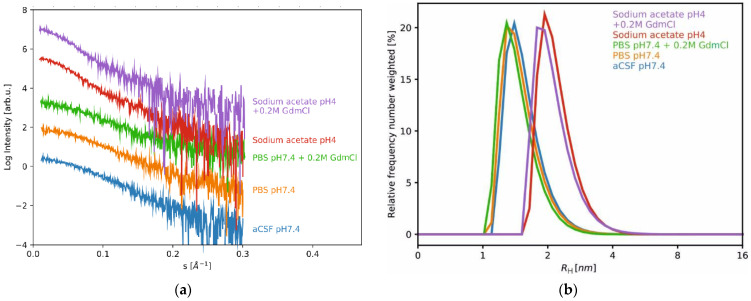
SAXS and DLS data collected for human cystatin C in mild denaturing conditions and aCSF: (**a**) SAXS data recorded for HCC at pH 4 and pH 7.4 and in the presence of 0.2 M GdmCl; (**b**) particle size distribution of human cystatin C (HCC), with the effect of 0.2 M GdmCl.

The same phenomenon was observed when the hydrodynamic radius (*R_H_*) was analyzed using the dynamic light scattering (DLS) method (Figure 3, Table 1). At pH 2.2 and pH 3, the HCC samples were characterized by *R_H_* values of 3.16 nm and 3.21 nm, respectively. Increases in pH to 4 and 5 led to decreased *R_H_* values, as follows: 2.22 and 2.15 nm in sodium acetate (without and with 0.2 M GdmCl, respectively) and 2.44 nm in citric acid at pH = 5. In the pH range from 6 to 10, the *R_H_* values varied from 1.47 nm to 1.63 nm, with the lowest value obtained in PBS with GdmCl and the highest in artificial cerebrospinal fluid (aCSF), both solutions were at pH 7.4. These observations indicated the presence of higher forms of HCC at lower pH levels, as also demonstrated by the SAXS method. The particle size distributions of HCC in all the mentioned pH conditions are presented in Figure 3. Under the tested conditions, three particle size populations can be distinguished, which after the SAXS analysis were classified as the monomer, dimer and tetramer. We also observed that the addition of 0.2 M GdmCl did not affect the size or shape of the HCC molecules. The SAXS and DLS data collected for human cystatin C in mild denaturing conditions and aCSF are presented in Table 1 and Figure 4. The addition of GdmCl to the acidic buffer (sodium acetate) admittedly led to an increase in *R_G_* and *D*_max_ values but not *R_H,_* while the addition of GdmCl to neutral buffer (PBS) led to a slight decrease in *R_G_* and *D*_max_ values but analogously not for *R_H_.*

### 2.2. Characterization of Oligomeric Species Present in HCC Solution at pH 3

**Figure 5:**
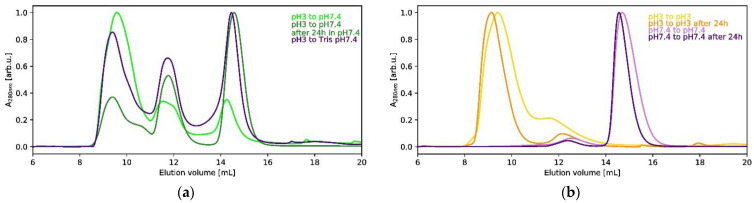
The results of the SEC analysis of the formation and stability of the tetrameric HCC form: (**a**) SEC elution profiles for HCC at pH 3 and transferred to pH 7.4 (PBS and Tris buffers); (**b**) SEC elution profiles for HCC at pH 3 transferred to pH 3 and pH 7.4 (PBS buffer). The chromatogram traces were normalized to value 1.

In order to characterize the oligomeric species present at pH 3, we performed size exclusion chromatography (SEC) of the HCC solution at 3 mg/mL. We observed an additional peak besides the two peaks characteristic of monomeric and dimeric species. The peak corresponding to the oligomeric form was concentrated and the SAXS curve was recorded for 2 h. The results of the SEC analysis, also including a stability test (24 h), are visualized in Figure 5.

The tetrameric form of HCC is characterized by a *R_G_* of 3.55 nm and *D*_max_ = 13.8 nm. A comparison of the I(0) value for the tetrameric HCC with the scattering of the molecular weight standard (bovine serum albumin, BSA) resulted in an MW for tetrameric HCC of 53 kDa, which clearly indicates the existence of the HCC tetramer in the solution.

In order to test the stability of the obtained tetramer, we ran a similar experiment; however, the isolated HCC tetramer was prepared at pH 3 and separated via SEC in PBS buffer at pH 7.4 or 20 mM Tris at pH 7.4 with 50 mM NaCl. The SAXS data were collected just after purification. We observed that during the first 10 min of SAXS measurement in PBS buffer, the *R_G_* value was 3.52 nm, but after 20 min it had decreased to 3.16 nm. During the SAXS measurements of HCC in Tris buffer, the *R_G_* and *D*_max_ values (Table 2) were stable and were equal to 2.71 nm and 12.5 nm, respectively. This indicated that the tetrameric form is unstable at physiological pH.

**Figure 6:**
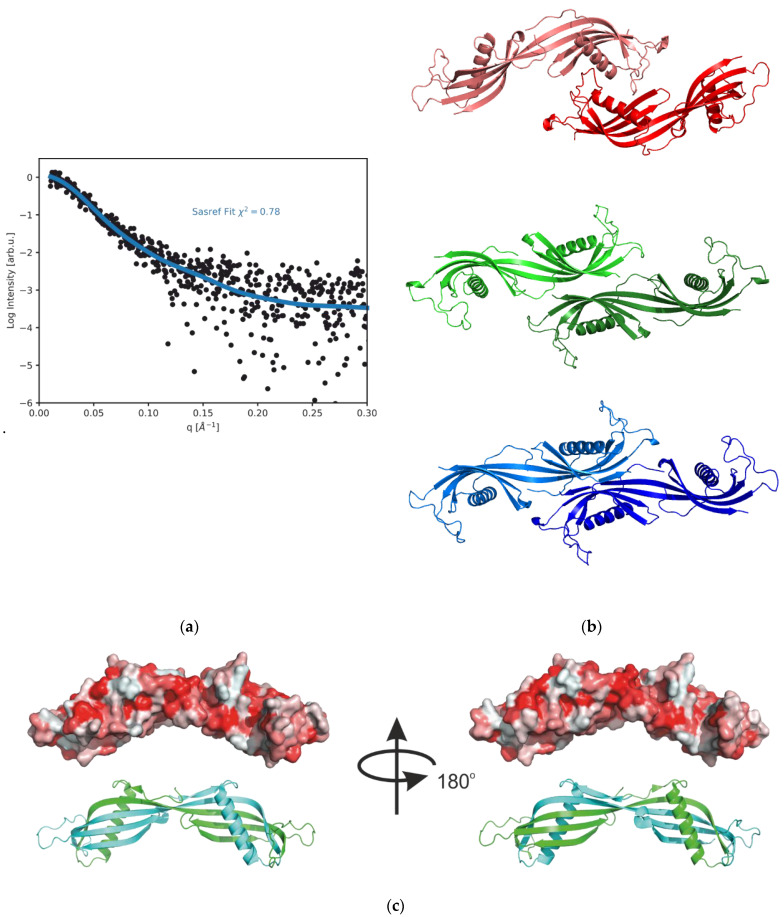
Model of tetrameric HCC obtained via rigid body modeling using the structure of the HCC dimer [10] and SAXS data: (**a**) the fit of the model to the experimental data for the selected SASREF run with the highest score; (**b**) three top models of the HCC tetramer obtained using SASREF; (**c**) the protein surface hydrophobicity (the more red color indicates higher hydrophobicity) (bottom) calculated for the domain-swapped HCC dimer.

Taking into account that the HCC tetramer is unstable at pH 7.4, we assume that it is formed by the association of two domain-swapped dimers. In order to model the structure of the tetrameric form of HCC, we used the dimeric structure of the HCC (PDB code: 1TIJ) and performed rigid body modeling using the SASREF program (25 runs). The majority of the runs gives a very good fit to the experimental data. An exemplary fit to the experimental data is shown in Figure 6a. Three best models, characterized by χ^2^ = 0.78, are presented in Figure 6b. All obtained models exhibit the elongated structure, where the ends of the domain-swapped HCC dimer interact with each other, although the resolution of the SAXS data cannot clearly be used to decipher the mode of interaction. From the three models with highest χ^2^ values, two of them consist of an HCC dimer interacting via a β sheet fragment that creates the cleft for the α-helix. In the third model with the highest score, the HCC dimers interact with each other via the α-helical fragment and one of the terminal loops.

**Figure 7:**
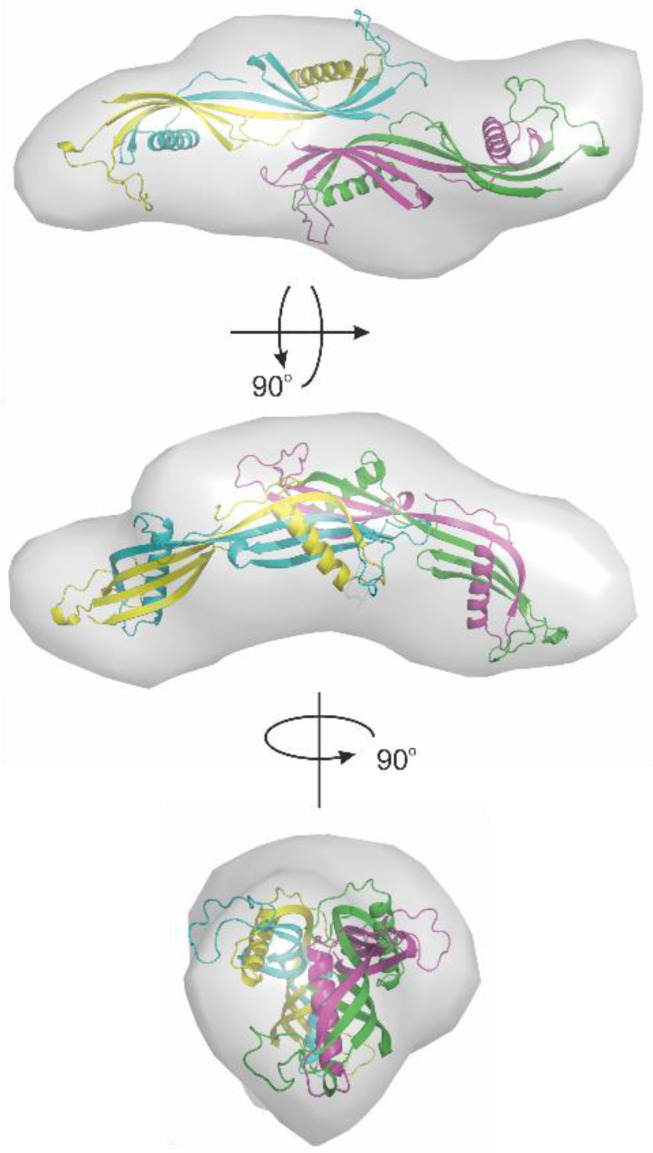
Low-resolution model of tetrameric HCC obtained using DAMMIF modeling and SAXS data collected at pH 3.0.

The SAXS data obtained for the HCC tetramer (pH = 3) were used for the calculation of a low-resolution model using DAMMIF. This structure is presented in Figure 7. The reconstructed molecular envelope exhibited a similar shape to that of the structure obtained using SASREF.

Using crystallographic models of the HCC monomer and dimer (Figure 6c) and the obtained model of the tetrameric form of HCC, it was possible to calculate the contributions of individual oligomeric forms to all scattering curves (pH 2.2–10) using the OLIGOMER program. These data are summarized in Table 3, where the characteristic pH-dependent behavior of HCC is evident. At low pH values (2.2, 3.0), only the tetrameric form of HCC is present.

## 3. Discussion

In this study, we analyzed the structure of HCC and its oligomeric forms using small-angle X-ray scattering and dynamic light scattering (hydrodynamic diameter) in solutions imitating various body fluids, commonly used buffers in HCC oligomerization studies and potentially useful Good’s buffers [24] with different pH levels.

Therefore, it should be assumed that the HCC dimer produced as a result of the domain-swapping phenomenon is the most stable form of small HCC oligomers. We suspect that the dimer formation is a kinetic limiting factor in the oligomerization process. This suggestion is consistent with the applied methodology, taking into account guanidinium chloride and other destabilizing factors, and potentially it can be extrapolated for the agents that cause amyloidosis.

The exact mechanism of the formation of oligomers and human cystatin C fibrils is still elusive. Despite the many studies with quite similar methodologies, different research groups have come to different, often contradictory conclusions, considering the domain-swapping phenomenon as either a crucial factor [25] or completely uninvolved in fibril formation [26].

Wahlbom and co-workers [25] described donut-shaped oligomers formed by wild-type and L68Q (highly amyloidogenic) HCC upon incubation of the monomeric proteins. They postulated that three-dimensional domain swapping is involved in the formation of the oligomers. Moreover, they suggested that the higher HCC structures are formed by the propagated domain swapping rather than by the assembly of domain-swapped cystatin C dimers, although this phenomena was discussed in the context of bigger (200–300 kDa) forms only. Using analogous methods of oligomerization (50 mM sodium acetate, 100 mM NaCl, pH 4), in 2017 Perlenfein et al. [26] observed that the V57N variant of HCC (described as resistant to dimerization or fibril formation) does not undergo domain swapping but still forms fibrils. This leads to the conclusion that domain swapping is not necessary for fibril formation. In 2013, Östner et al. [18] concluded that oligomers are highly ordered, domain-swapped assemblies of cystatin C and that these oligomers could not build larger oligomers without domain swapping. In this study, they obtained dimers, trimers, tetramers and dodecamers but observed no other combinations (e.g., pentamers, hexamers, heptamers, octamers or nonamers). Thus, it is necessary to consider whether domain swapping is a general mechanism of amyloid aggregation or whether other mechanisms also exist. In 2004 [27], the transformation of monomeric cystatin C in amyloid fibrils at a very low pH of 2.0 was reported, but no molecular intermediates of this transformation were observed, while the SAXS and DLS results presented in this paper clearly indicate the presence at pH 2.2 of tetrameric HCC (without incubation). Dimerization via domain swapping is not restricted to HCC; later studies of human cystatin E properties also indicated that the domain-swapped dimer is comparable to HCC [28].

Shingate et al. (2015) [29] analyzed several protein structures in which domain swapping was observed, especially focusing on interface zones, and emphasized that it is necessary to weaken the domain-swapped interface (DSI) region in order to create an oligomer with swapped domains. Moreover, in over 80% of the tested dataset of analyzed proteins, they noted that the domain swapping mechanism was observed in an acidic environment; however, they summarized that the size of the analyzed pool of structures was too small for the general conclusion that low pH promotes the domain swapping mechanism. In 2014, Baler and co-workers [30], using molecular dynamics, found that low pH, which contributes to the oligomerization of human serum albumin (HSA), leads to the exposure of core hydrophobic regions that are critically involved in electrostatic repulsion. The observed HSA conformation change was reversible and drastic, similar to the case of HCC, showing a change in the protein structure without prior incubation. Moreover, they noted that the change to acidic pH decreased the HSA denaturation temperature, which led to the conclusion that the pH can significantly change the conditions of the protein oligomerization [30]. In 2020, Santos et al. [31], assuming that the solvent conditions impact on the solubility of proteins, proposed an algorithm predicting the intrinsically disordered protein structure based on the pH. They expected that the pH-dependent protein aggregation is determined by the charge and lipophilicity. Interestingly, they were able to predict the assembly and disassembly of the fibrils when changing the pH [31]. Therefore, it seems obvious that the buffer composition and ionic strength may also play a significant role in the oligomerization of proteins. There are several reports devoted to this issue, including the work by Batoulis et al. [32], who showed the importance of the concentration of divalent ions in this process, or Samantray and co-workers [33], who focused on the ionic strength in a model system based on glycosaminoglycans. However, this issue was not directly investigated in this manuscript, as we focused on buffers with close to physiological ionic strength that could potentially be used in HCC oligomerization studies. Moreover, we did not observe any influence of the presence divalent ions (at the used concentrations) on the HCC form, although we observed a small increase in all studied parameters (*R*_G_, *D*_max_ and *R*_H_) of HCC in aCSF, containing both Ca^2+^ and Mg^2+^. Based on the obtained data, we can conclude that the observed formation of dimeric and tetrameric forms was clearly pH-dependent.

Therefore, it can be assumed that the tetrameric forms of human cystatin C that we have noted may arise through the association of domain-swapped dimers, possibly without further domain swapping. This type of association is supported by the relatively low stability of these structures, which decayed into dimers after changing the pH.

## 4. Materials and Methods

### 4.1. Protein Expression and Purification

The heterologous HCC expression was carried out in SHuffle^®^ T7-competent *E. coli* cells (New England Biolabs, Ipswich, MA, USA) using a modified protocol [34]. The bacteria were grown in an LB standard medium (10 g/L of tryptone, 5 g/L of yeast extract and 10 g/L of NaCl) (BioShop Canada, Burlington, ON, Canada) with kanamycin (50 µg/mL) ((BioShop Canada) at 30 °C, with shaking at 180 rpm (Lab Companion, ISS-4075R). The induction process was performed in the exponential growth phase via the addition of 0.4 mM of IPTG (BioShop, Mainway, Burlington, ON, Canada) with subsequent incubation for 4 h at 30 °C, with shaking at 180 rpm. The bacteria were then centrifuged for 15 min at 3000× *g* and 4 °C and washed with cold water (MQ-grade). The pellet was frozen in liquid nitrogen and stored at −80 °C until the protein extraction phase. In the next step, the pellet from 2 L of bacterial culture was carefully thawed out, resuspended in 20 mL of cold buffer A (20 mM Tris-HCl pH 7.5) (BioShop, Mainway, Burlington, ON, Canada), with 1 mM benzamidinium chloride (BZA) (BioShop, Mainway, Burlington, ON, Canada), drawn through a needle three times and then sonicated (55% amplitude, 15 × 30 s, with 30 s interval between each sonication pulse, 4 °C; Vibra-Cell™, Sonics, Newtown, CT, USA). Next, the sample was centrifuged for 40 min at 40,000× *g* and 4 °C. The supernatant passed through a 0.45 µm filter was loaded onto the Äkta PURE 25 FPLC system (GE Healthcare, Barrington, IL, USA) equilibrated with buffer A with BZA. The sample was injected onto combined 2 × 1 mL Sepharose Q (HiTrap Q FF, GE Healthcare, Barrington, IL, USA) and 1 × 5 mL Sepharose S (HiTrap SP FF, GE Healthcare, Barrington, IL, USA) columns. The Sepharose Q columns were removed before washing. The elution process was performed with increasing concentrations of NaCl in buffer B (20 mM of Tris-HCl at pH 7.5, 1 M of NaCl) with 1 mM of BZA and was monitored via absorbance spectroscopy at 280 nm. A typical chromatogram image at this step showed two high peaks. The presence of HCC in the subsequent fractions was each time confirmed via SDS page electrophoresis. The fractions containing approximately 14 kDa proteins were then dialyzed overnight against 20 mM of ammonium bicarbonate at pH 8.0. The next day, around 14 mL of sample was concentrated to 2 mL using an Amicon Ultra-15 Centrifugal Filter Unit (#UFC9010, Millipore, Burlington, MA, USA) and loaded onto a HiLoad^®^ Superdex 75 10/300 GL column (GE Healthcare, Barrington, IL, USA) equilibrated with 50 mM of ammonium bicarbonate (Sigma Aldrich, Saint Louis, MO, USA) at pH 8.0. Afterwards, the SEC procedure fractions containing monomeric HCC were connected and lyophilized. The purity of the preparation was checked via SDS page electrophoresis.

### 4.2. Protein Oligomerization

To analyze the structure and stability of HCC oligomers, lyophilized HCC was prepared in citric acid at pH 3 and then transferred using size exclusion chromatography (SEC) to buffers of different pH and NaCl concentrations. Each time, before the SEC procedure, the sample was centrifuged for 20 min at 20,000× *g* and 4 °C. The sample, which was transferred to 20 mM of Tris and 50 mM of NaCl at pH 7.4, was prepared in citric acid at pH 3 (50 mM of citric acid, 100 mM of NaCl) and without incubation was separated on a HiLoad^®^ Superdex 75 10/300 GL column (GE Healthcare, Barrington, IL, USA). The sample, which was transferred to PBS at pH 7.4, was prepared in citric acid at pH 3 with 0.2 M of GdmCl, incubated for 20 h at 47 °C and 700 rpm (ThermoMixer C, Eppendorf, Hamburg, Germany) and separated on a HiLoad^®^ Superdex 200 Increase 10/300 GL column (GE Healthcare, Barrington, IL, USA). The sample, which was transferred to citric acid at pH 3, was prepared analogously. To analyze the HCC oligomerization from the monomer and dimer, SAXS measurements were performed. The protein taken for analysis came directly from the SEC procedure on the HiLoad^®^ Superdex 75 10/300 GL column (GE Healthcare, Barrington, IL, USA) equilibrated with 50 mM of ammonium bicarbonate at pH 8.0, described in detail in Section 4.1. The SAXS measurements were carried out every 10 min for 9 h for the HCC monomer and for 20 h for the HCC in dimeric state. In order to test the stability of the HCC tetramer at pH 3, the HCC sample was prepared at pH 3 and 1 mL of the solution was run on the HiLoad^®^ Superdex 75 10/300 GL column (GE Healthcare, Barrington, IL, USA) equilibrated with citric acid at pH 3 or PBS buffer. The fractions containing HCC were collected and incubated at 4 °C for 24 h. The fractions were concentrated using an Amicon Ultra-15 Centrifugal Filter Unit (# UFC9010, Millipore) and run once again on the HiLoad^®^ Superdex 75 10/300 GL column (GE Healthcare, Barrington, IL, USA) equilibrated with citric acid at pH 3 or PBS buffer. In the control experiments, the HCC sample dissolved in PBS was run on the SEC column in the same manner as for the sample at pH 3.

### 4.3. Dynamic Light Scattering

Lyophilized HCC was dissolved in ice-cold water (MQ grade) and centrifuged for 20 min at 20,000× *g* and 4 °C. Just before taking the measurements, the sample was mixed to 1:1 with twice-concentrated buffer (Table 4). The final protein concentration was in the range of 3.3–3.4 mg/mL. The same samples were simultaneously measured using SAXS. The dynamic light experiments for all HCC samples were conducted using a Litesizer 500 instrument with Caliope software (Anton Paar, Graz, Austria). The HCC solutions were placed in quartz cuvettes (sample volume 50 µL). The measurements were carried out at 25 °C and an angle of 90° in quick mode (30 × 10 s) in at least 4 replicates.

### 4.4. Small-Angle X-ray Scattering

The solution scattering data for the studied oligomeric forms of human cystatin C were obtained with the use of the XEUSS 2.0 laboratory system (XENOCS, Grenoble, France) operated using an X-ray beam (λ = 1.34 Å) generated by a MetalJet D2+ (Excillium, Kista, Sweden) microfocus X-ray source with a gallium target and focused using 2D multilayer optics. The SAXS data were recorded in the momentum transfer range of 0.01 < s < 0.3 Å^−1^, where s = 4πsinθλ^−1^ at a temperature of 10 °C. The HCC samples were injected into the low-noise flow cell (XENOCS, France) and exposed for 600 s per single frame, and at least 10 independent scattering images were collected for each sample. The working HCC concentration was 3 mg/mL, and for each sample an identical number of frames for the appropriate buffer was also collected. The SAXS patterns were processed using FOXTROT [35] and PRIMUS [36] computer programs from the ATSAS package (version 3.1.1) [37].

### 4.5. SAXS Data Analysis

The scattering data collected for HCC under different conditions were used for a comparison with the experimental structures of the HCC monomer (PDB code: 3GAX [8]) and domain-swapped dimer in extended conformation (PDB code: 1TIJ [10]). The theoretical scattering profiles of HCC were calculated and fitted to the SAXS data using the above mentioned structures and CRYSOL [38]. An analysis of the particular oligomeric states of HCC on the basis of SAXS data was performed using the methodology previously described for the domain-swapping process of HCC induced using synchrotron radiation [23] and the OLIGOMER program from the ATSAS package [37]. The structure of the HCC tetramer was modeled using SASREF [39]. Twenty-five independent runs of SASREF with assumed P2 symmetry were performed using the HCC domain-swapped dimer (PDB code: 1TIJ). Ten models with the best fit (lowest χ^2^) to the experimental data were then chosen for the further analysis. Independently, the low-resolution model of the HCC tetramer was also generated using the DAMMIF [40,41] program from the ATSAS online package (https://www.embl-hamburg.de/biosaxs/atsas-online/dammif, accessed on 29 September 2022) with assumed P2 symmetry and by averaging 20 individually obtained models in DAMAVER. For visualizations, the bead model was converted into the map in UCSF Chimera and visualized in PyMOL (PyMOL Molecular Graphics System 2.1, Schrödinger, LLC, NY, USA). The model of the HCC tetramer was fitted into the map using the UCFS Chimera Fit in Map procedure (UCSF Chimera 1.15, University of California, CA, USA) [42]. The secondary structure models were also visualized using Mol*Viewer [43].

## 5. Conclusions

The human cystatin C oligomeric forms are highly dependent on the pH of the buffer. Both the SAXS and DLS methods allow the assessment of the HCC oligomerization state. Oligomerization was observed in the range of pH 2.2–5. No influence of 0.2 M of guanidinium chloride on the HCC structure was observed. The obtained tetrameric form is unstable after exchange to a neutral pH. The HCC tetramer is formed via the association of domain-swapped dimers.

## Figures and Tables

**Table 1:** The structural parameters characterizing HCC in different conditions. The radii of gyration (*R_G_*) and the size of the molecule *D*_max_ were calculated from the SAXS data and the hydrodynamic radii (*R_H_*) from the DLS results.

Sample	*R*_G_ [nm]	*D*_max_ [nm]	*R*_H_ [nm]
Glycine pH 2.2	3.91 ± 0.1	17.0	3.16 ± 0.1
Citric acid pH 3	3.42 ± 0.1	14.0	3.21 ± 0.06
Sodium acetate pH 4	2.64 ± 0.05	12.0	2.22 ± 0.06
Sodium acetate pH 4 + GdmCl	2.98 ± 0.08	12.5	2.15 ± 0.06
Citric acid pH 5	2.41 ± 0.07	11.2	2.47 ± 0.15
MES pH 6	1.55 ± 0.04	6.0	1.50 ± 0.05
Citric acid pH 7	1.64 ± 0.05	6.7	1.51 ± 0.02
aCSF pH 7.4	1.76 ± 0.03	8.0	1.63 ± 0.05
PBS pH 7.4	1.63 ± 0.03	6.3	1.53 ± 0.05
PBS pH 7.4 + GdmCl	1.63 ± 0.12	6.6	1.47 ± 0.05
Tricine pH 8.8	1.68 ± 0.07	6.2	1.48 ± 0.05
Glycine pH 10	1.73 ± 0.05	7.8	1.57 ± 0.07

**Table 2:** The analysis of HCC oligomeric states at pH 3.0 and 7.4. The SAXS data fitting analysis using a mixture of a monomer, dimer and tetramer; pH 3 to pH 7.4 (PBS, First 10 min)—first 10 min of measurement, sample prepared in citric acid pH 3 and transferred to PBS at pH 7.4 with SEC; pH 3 to pH 7.4 (PBS, 20–1 h 20 min), average of next 11 frames covering 1 h and 10 min of measurement; pH 3 to pH 7.4 (20 mM Tris, 50 mM NaCl), sample prepared in citric acid at pH 3 and transferred to Tris buffer at pH 7.4 with SEC; pH 3 to pH 3—sample prepared in citric acid at pH 3 after SEC to the same buffer.

Sample	Monomer	Dimer	Tetramer
pH 3 to pH 7.4 (PBS, First 10 min)	0.07	0.00	0.92
pH 3 to pH 7.4 (PBS, 20–1 h 20 min)	0.00	1.00	0.00
pH 3 to pH 7.4 (20 mM Tris, 50 mM NaCl)	0.00	1.00	-
pH 3 to pH 3	0.00	0.00	0.99

**Table 3:** The fractions of oligomeric forms of HCC calculated from SAXS data.

Sample	Monomer	Dimer	Tetramer
Glycine pH 2.2	0.00	0.00	1.00
Citric acid pH 3	0.00	0.00	1.00
Sodium acetate pH 4	0.00	0.85	0.15
Sodium acetate pH 4 + GdmCl	0.00	0.70	0.30
Citric acid pH 5	0.07	0.93	-
MES pH 6	0.97	0.03	-
Citric acid pH 7	0.84	0.16	-
aCSF pH 7.4	0.83	0.17	-
PBS pH 7.4	0.89	0.10	-
PBS pH 7.4 + GdmCl	0.93	0.00	0.07
Tricine pH 8.8	0.87	0.13	-
Glycine pH 10	0.85	0.15	-

**Table 4:** The chemical compositions of the buffers and solutions used in all experiments.

Buffer	Final Buffer Composition ^1^	pH ^2^
Glycine pH 2.2	50 mM Glycine, 100 mM NaCl	2.2
Citric acid pH 3	50 mM Citric acid, 100 mM NaCl	3
Sodium acetate pH 4	50 mM Sodium acetate, 100 mM NaCl	4
Sodium acetate pH 4 + GdmCl	50 mM Sodium acetate, 100 mM NaCl, 0.2 M guanidinium chloride	4
Citric acid pH 5	50 mM Citric acid, 100 mM NaCl	5
MES pH 6	50 mM MES, 100 mM NaCl	6
Citric acid pH 7	50 mM Citric acid, 100 mM NaCl	7
aCSF pH 7.4	5.4 mM KCl, 5 mM Hepes, 1.8 mM CaCl_2_, 1 mM MgCl_2_, 135 mM NaCl	7.4
PBS pH 7.4	2.7 mM KCl, 1.8 mM KH_2_PO_4_, 0.9 mM Na_3_PO_4_, 137 mM NaCl ^3^	7.4
PBS pH 7.4 + GdmCl	2.7 mM KCl, 1.8 mM KH_2_PO_4_, 0.9 mM Na_3_PO_4_, 137 mM NaCl ^3^, 0.2 M guanidinium chloride	7.4
Tricine pH 8.8	50 mM Tricine, 100 mM NaCl	8.8
Glycine pH 10	50 mM Glycine, 100 mM NaCl	10

^1^ Glycine, MES—Sigma Aldrich, Saint Louis, MO, USA; Citric acid, Calcium chloride, Sodium acetate—POCH, Gliwice, Poland; PBS, Tricine, Sodium chloride—BioShop Canada, Burlington, Canada; HEPES, Guanidinium hydrochloride—Carl Roth GmbH + Co. KG, Karlsruhe, Germany; Magnesium chloride—Merck, Darmstadt, Germany. ^2^ The pH was adjusted with HCl (acidic buffers) or NaOH (basic buffers). ^3^ PBS prepared from tablets (BioShop Canada, #PBS404.100). Values calculated based on the manufacturer’s data.

## Data Availability

All data generated in this study are presented in the article. The raw data are available on request from the corresponding author.

## References

[1] Grubb A., Löfberg H. (1982). Human Gamma-Trace, a Basic Microprotein: Amino Acid Sequence and Presence in the Adenohypophysis. Proc. Natl. Acad. Sci. USA.

[2] Brzin J., Popovič T., Turk V., Borchart U., Machleidt W. (1984). Human Cystatin, a New Protein Inhibitor of Cysteine Proteinases. Biochem. Biophys. Res. Commun..

[3] Abrahamson M., Mason R.W., Hansson H., Buttle D.J., Grubb A., Ohlsson K. (1991). Human Cystatin C. Role of the *N* -Terminal Segment in the Inhibition of Human Cysteine Proteinases and in Its Inactivation by Leucocyte Elastase. Biochem. J..

[4] Abrahamson M., Ritonja A., Brown M.A., Grubb A., Machleidt W., Barrett A.J. (1987). Identification of the Probable Inhibitory Reactive Sites of the Cysteine Proteinase Inhibitors Human Cystatin C and Chicken Cystatin. J. Biol. Chem..

[5] Janowski R., Kozak M., Jankowska E., Grzonka Z., Grubb A., Abrahamson M., Jaskolski M. (2001). Human Cystatin C, an Amyloidogenic Protein, Dimerizes through Three-Dimensional Domain Swapping. Nat. Struct Biol..

[6] Kozak M., Jankowska E., Janowski R., Grzonka Z., Grubb A., Alvarez Fernandez M., Abrahamson M., Jaskolski M. (1999). Expression of a Selenomethionyl Derivative and Preliminary Crystallographic Studies of Human Cystatin C. Acta Cryst. D Biol. Cryst..

[7] Janowski R., Abrahamson M., Grubb A., Jaskolski M. (2004). Domain Swapping in N-Truncated Human Cystatin C. J. Mol. Biol..

[8] Kolodziejczyk R., Michalska K., Hernandez-Santoyo A., Wahlbom M., Grubb A., Jaskolski M. (2010). Crystal Structure of Human Cystatin C Stabilized against Amyloid Formation. FEBS J..

[9] Maszota-Zieleniak M., Jurczak P., Orlikowska M., Zhukov I., Borek D., Otwinowski Z., Skowron P., Pietralik Z., Kozak M., Szymańska A. (2020). NMR and Crystallographic Structural Studies of the Extremely Stable Monomeric Variant of Human Cystatin C with Single Amino Acid Substitution. FEBS J..

[10] Janowski R., Kozak M., Abrahamson M., Grubb A., Jaskolski M. (2005). 3D Domain-Swapped Human Cystatin C with Amyloidlike Intermolecular Beta-Sheets. Proteins.

[11] Åkesson A., Lindström V., Nyman U., Jonsson M., Abrahamson M., Christensson A., Björk J., Grubb A. (2020). Shrunken Pore Syndrome and Mortality: A Cohort Study of Patients with Measured GFR and Known Comorbidities. Scand. J. Clin. Lab. Investig..

[12] Grubb A. (2020). Shrunken Pore Syndrome—A Common Kidney Disorder with High Mortality. Diagnosis, Prevalence, Pathophysiology and Treatment Options. Clin. Biochem..

[13] Ghiso J., Jensson O., Frangione B. (1986). Amyloid Fibrils in Hereditary Cerebral Hemorrhage with Amyloidosis of Icelandic Type Is a Variant of Gamma-Trace Basic Protein (Cystatin C). Proc. Natl. Acad. Sci. USA.

[14] Palsdottir A., Snorradottir A.O., Thorsteinsson L. (2006). Hereditary Cystatin C Amyloid Angiopathy: Genetic, Clinical, and Pathological Aspects. Brain Pathol..

[15] Ólafsson Í., Thorsteinsson L., Jensson Ó. (1996). The Molecular Pathology of Hereditary Cystatin C Amyloid Angiopathy Causing Brain Hemorrhage. Brain Pathol..

[16] Graffagnino C., Herbstreith M.H., Schmechel D.E., Levy E., Roses A.D., Alberts M.J. (1995). Cystatin C Mutation in an Elderly Man with Sporadic Amyloid Angiopathy and Intracerebral Hemorrhage. Stroke.

[17] Mathews P.M., Levy E. (2016). Cystatin C in Aging and in Alzheimer’s Disease. Ageing Res. Rev..

[18] Östner G., Lindström V., Hjort Christensen P., Kozak M., Abrahamson M., Grubb A. (2013). Stabilization, Characterization, and Selective Removal of Cystatin C Amyloid Oligomers. J. Biol. Chem..

[19] Chrabąszczewska M., Sieradzan A.K., Rodziewicz-Motowidło S., Grubb A., Dobson C.M., Kumita J.R., Kozak M. (2020). Structural Characterization of Covalently Stabilized Human Cystatin C Oligomers. Int. J. Mol. Sci..

[20] Chrabąszczewska M., Maszota-Zieleniak M., Pietralik Z., Taube M., Rodziewicz-Motowidło S., Szymańska A., Szutkowski K., Clemens D., Grubb A., Kozak M. (2018). Cyclic Trimer of Human Cystatin C, an Amyloidogenic Protein—Molecular Dynamics and Experimental Studies. J. Appl. Phys..

[21] Da Vela S., Svergun D.I. (2020). Methods, Development and Applications of Small-Angle X-Ray Scattering to Characterize Biological Macromolecules in Solution. Curr. Res. Struct. Biol..

[22] Jacques D.A., Trewhella J. (2010). Small-Angle Scattering for Structural Biology-Expanding the Frontier While Avoiding the Pitfalls: Small-Angle Scattering for Structural Biology. Protein Sci..

[23] Taube M., Pietralik Z., Szymanska A., Szutkowski K., Clemens D., Grubb A., Kozak M. (2019). The Domain Swapping of Human Cystatin C Induced by Synchrotron Radiation. Sci. Rep..

[24] Good N.E., Winget G.D., Winter W., Connolly T.N., Izawa S., Singh R.M.M. (1966). Hydrogen Ion Buffers for Biological Research. Biochemistry.

[25] Wahlbom M., Wang X., Lindström V., Carlemalm E., Jaskolski M., Grubb A. (2007). Fibrillogenic Oligomers of Human Cystatin C Are Formed by Propagated Domain Swapping. J. Biol. Chem..

[26] Perlenfein T.J., Mehlhoff J.D., Murphy R.M. (2017). Insights into the Mechanism of Cystatin C Oligomer and Amyloid Formation and Its Interaction with β-Amyloid. J. Biol. Chem..

[27] Nilsson M., Wang X., Rodziewicz-Motowidlo S., Janowski R., Lindström V., Önnerfjord P., Westermark G., Grzonka Z., Jaskolski M., Grubb A. (2004). Prevention of Domain Swapping Inhibits Dimerization and Amyloid Fibril Formation of Cystatin C. J. Biol. Chem..

[28] Dall E., Hollerweger J.C., Dahms S.O., Cui H., Häussermann K., Brandstetter H. (2018). Structural and Functional Analysis of Cystatin E Reveals Enzymologically Relevant Dimer and Amyloid Fibril States. J. Biol. Chem..

[29] Shingate P., Warwicker J., Sowdhamini R. (2015). Energetic Calculations to Decipher PH-Dependent Oligomerization and Domain Swapping of Proteins. PLoS ONE.

[30] Baler K., Martin O.A., Carignano M.A., Ameer G.A., Vila J.A., Szleifer I. (2014). Electrostatic Unfolding and Interactions of Albumin Driven by PH Changes: A Molecular Dynamics Study. J. Phys. Chem. B.

[31] Santos J., Iglesias V., Santos-Suárez J., Mangiagalli M., Brocca S., Pallarès I., Ventura S. (2020). PH-Dependent Aggregation in Intrinsically Disordered Proteins Is Determined by Charge and Lipophilicity. Cells.

[32] Batoulis H., Schmidt T.H., Weber P., Schloetel J.-G., Kandt C., Lang T. (2016). Concentration Dependent Ion-Protein Interaction Patterns Underlying Protein Oligomerization Behaviours. Sci. Rep..

[33] Samantray S., Olubiyi O.O., Strodel B. (2021). The Influences of Sulphation, Salt Type, and Salt Concentration on the Structural Heterogeneity of Glycosaminoglycans. Int. J. Mol. Sci..

[34] Tovar-Anaya D.O., Vera-Robles L.I., Vieyra-Eusebio M.T., García-Gutiérrez P., Reyes-Espinosa F., Hernández-Arana A., Arroyo-Reyna J.A., Zubillaga R.A. (2019). Stabilized Human Cystatin C Variant L47C/G69C Is a Better Reporter Than the Wild-Type Inhibitor for Characterizing the Thermodynamics of Binding to Cysteine Proteases. Protein J.

[35] Girardot R., Viguier G., Pérez J., Ounsy M. FOXTROT: A JAVA-Based Application to Reduce and Analyse SAXS and WAXS Piles of 2D Data at Synchrotron SOLEIL, Synchrotron Soleil, Saint-Aubin, France. Proceedings of the canSAS-VIII.

[36] Konarev P.V., Volkov V.V., Sokolova A.V., Koch M.H.J., Svergun D.I. (2003). *PRIMUS*: A Windows PC-Based System for Small-Angle Scattering Data Analysis. J. Appl. Cryst..

[37] Manalastas-Cantos K., Konarev P.V., Hajizadeh N.R., Kikhney A.G., Petoukhov M.V., Molodenskiy D.S., Panjkovich A., Mertens H.D.T., Gruzinov A., Borges C. (2021). *ATSAS 3.0*: Expanded Functionality and New Tools for Small-Angle Scattering Data Analysis. J. Appl. Cryst..

[38] Svergun D., Barberato C., Koch M.H.J. (1995). *CRYSOL*—A Program to Evaluate X-Ray Solution Scattering of Biological Macromolecules from Atomic Coordinates. J. Appl. Cryst..

[39] Petoukhov M.V., Svergun D.I. (2005). Global Rigid Body Modeling of Macromolecular Complexes against Small-Angle Scattering Data. Biophys. J..

[40] Volkov V.V., Svergun D.I. (2003). Uniqueness of Ab Initio Shape Determination in Small-Angle Scattering. J. Appl. Cryst..

[41] Franke D., Svergun D.I. (2009). *DAMMIF,* a Program for Rapid *Ab-Initio* Shape Determination in Small-Angle Scattering. J. Appl. Cryst..

[42] Pettersen E.F., Goddard T.D., Huang C.C., Couch G.S., Greenblatt D.M., Meng E.C., Ferrin T.E. (2004). UCSF Chimera? A Visualization System for Exploratory Research and Analysis. J. Comput. Chem..

[43] Sehnal D., Bittrich S., Deshpande R., Svobodová R., Berka K., Bazgier V., Velankar S., Burley S.K., Koča J., Rose A.S. (2021). Mol* Viewer: Modern web app for 3D visualization and analysis of large biomolecular structures. Nucl. Acids Res..

